# *HCAR1* Modulates Ferroptosis in Gastric Cancer via Lactate-Mediated AMPK-SCD1 Signaling and Lipid Metabolism

**DOI:** 10.32604/or.2025.067247

**Published:** 2025-09-26

**Authors:** Songhua Bei, Qianqian Guo, Xinglei Wu, Fan Li, Yaya Xie, Xiaohong Zhang, Li Feng, Xingxing Zhang

**Affiliations:** 1 Fengxian District Center Hospital Graduate Student Training Base, Jinzhou Medical University, Shanghai, 201499, China; 2 Endoscopy Center, Minhang Hospital, Fudan University, Shanghai, 201100, China; 3 Department of Gastroenterology, Shanghai Jiaotong University Affiliated Sixth People’s Hospital South Campus, Shanghai, 201499, China

**Keywords:** Gastric cancer, lipid metabolism, ferroptosis, hydroxycarboxylic acid receptor 1 (HCAR1), lactate, AMP-activated protein kinase-stearoyl-CoA desaturase 1 (AMPK-SCD1) signaling

## Abstract

**Background:**

Ferroptosis is a type of regulated cell death characterized by iron-dependent lipid peroxidation, which has been linked to tumor progression and therapeutic resistance. However, the contribution of lactate metabolism and its receptor, hydroxycarboxylic acid receptor 1 (HCAR1), in ferroptosis regulation in gastric cancer (GC) remains poorly understood. Focusing specifically on its effects on cell proliferation, ferroptosis regulation, and the disruption of lactate-mediated metabolic pathways, the study aimed to clarify the role of HCAR1 in GC progression.

**Methods:**

Bioinformatics analysis identified prognostic genes associated with ferroptosis in GC. Receiver operating characteristic (ROC) curves were generated to assess the diagnostic potential of the predictive genes. The biological role of HCAR1 was investigated through gain and loss-of-function experiments in GC cell lines, followed by assessments of cell viability, oxidative stress indicators, gene/protein expression, and ferroptosis sensitivity under lactate stimulation or HCAR1 modulation.

**Results:**

HCAR1 was significantly upregulated in GC tissues and linked to poor patient outcomes. Silencing HCAR1 inhibited GC cell growth and induced ferroptosis, as shown by increased levels of reactive oxygen species (ROS) and malondialdehyde (MDA), along with decreased expression of solute carrier family 7 member 11 (SLC7A11) and glutathione peroxidase 4 (GPX4). Conversely, HCAR1 overexpression or exposure to extracellular lactate inhibited ferroptosis and activated antioxidant defenses. Mechanistically, lactate activation of HCAR1 increases ATP levels, which in turn inactivates AMP-activated protein kinase (AMPK). It also upregulates stearoyl-CoA desaturase 1 (SCD1) through the sterol regulatory element binding protein 1 (SREBP1) signaling pathway. Blocking HCAR1 reversed these effects and restored ferroptosis sensitivity.

**Conclusion:**

HCAR1 mediates lactate-driven ferroptosis resistance in GC through the AMPK-SCD1 signaling pathway. Targeting the HCAR1-lactate axis may offer a promising strategy for overcoming metabolic adaptation and improving GC treatment outcomes.

## 1 Introduction

Gastric cancer (GC), also referred to as stomach cancer, ranks as the fifth most prevalent malignancy and the fourth leading cause of cancer mortality globally [1]. About 95% of cases of GC are stomach adenocarcinoma [2], which is the most common kind [3]. Research has demonstrated that the highest incidence of GC occurs in East Asia, with relatively high rates also observed in Eastern and Central Europe [4]. GC development has been associated with *Helicobacter pylori* infection, diet-related influences, and genetic vulnerability [5]. Diagnosis is typically confirmed through upper gastrointestinal endoscopy with biopsy. Ferroptosis, an iron-dependent cell death mechanism characterized by lipid peroxidation, has gained increasing attention in recent research [6]. Research has demonstrated that Ophiopogonin B inhibits GC cell growth by inducing ferroptosis by downregulating *solute carrier family 7 member 11* (*SLC7A11*, also known as x*CT*) and *glutathione peroxidase 4* (*GPX4*) expression [7]. Emerging evidence suggests that ferroptosis is critically involved in the development of GC and may serve as a promising therapeutic target. Furthermore, numerous studies are exploring the impact of various signaling pathways on ferroptosis in GC cells. Thus, it is essential to comprehend the processes and regulatory functions of ferroptosis in GC to create novel treatment approaches.

As a newly identified form of regulated cell death, ferroptosis has garnered growing interest in the field of oncology. For instance, high doses of artesunate (ART) induce reactive oxygen species (ROS)-mediated DNA damage and cell death in ovarian cancer (OV) cells. This process is closely related to ferroptosis [8]. According to melanoma research, ferroptosis is negatively regulated by miR-137, which targets the glutamine transporter *SLC1A5*, and ferroptosis is promoted by decreased miR-137 expression [9]. *GPX4* inhibitors (1s, 3R)-RSL3, ML-162, as well as ART cause ferroptosis in head and neck cancer (HNC) cells, which is followed by the buildup of ROS and the depletion of glutathione (GSH) [10]. In addition, growing evidence indicates that biomarkers associated with ferroptosis contribute to both prognosis evaluation and therapeutic targeting in GC. For instance, ferroptosis induces GC cell death by promoting ROS accumulation, while *ACTL6A* inhibits ferroptosis by upregulating *GCLC*, thereby promoting the development of GC [11]. *SOX13* stimulates the formation of mitochondrial supercomplexes, enhances GC cell resistance to ferroptosis, and renders them therapeutically resistant [12]. *ATF2* promotes the malignant progression of GC cells by inhibiting sorafenib-induced ferroptosis and is associated with poor prognosis [13]. *BAP31* inhibits ferroptosis of GC cells by regulating *VDAC1* function. Its high expression not only promotes GC progression but may also become a potential therapeutic target [14]. Therefore, in-depth exploration of the key regulatory factors of ferroptosis is of great significance for exploring new therapeutic strategies.

*HCAR1* is primarily activated by lactate, a metabolic byproduct of glycolysis [15]. According to recent research, *HCAR1* may be involved in cancer metabolism. Enhanced glycolysis driven by the Warburg effect accounts for the elevated lactate concentrations frequently seen in cancer cells [16]. *HCAR1* may contribute to tumor development and progression through its regulation of lactate-mediated signaling pathways. For instance, Shi et al. reported that the curcumin analog NL01 suppressed the expression of *HCAR1* and monocarboxylate transporter 1 (*MCT1*), while activating the AMP-activated protein kinase (AMPK) pathway, ultimately triggering ferroptosis in OV cells via the sterol regulatory element-binding protein 1 (*SREBP1*) axis [17]. According to Zhao et al., lactate promotes ATP production via *MCT1* in hepatocellular carcinoma cells, suppressing AMPK and upregulating *SREBP1* and *SCD1*, which in turn increases their resistance to ferroptosis [18]. Inhibition of lactate absorption activated AMPK, decreased SCD1, and enhanced ferroptosis. Additionally, Xie et al. demonstrated that lactate upregulates *HCAR1* expression through the *Signal Transducer and Activator of Transcription 3* (*STAT3)* signaling pathway [19]. Lactate induces the formation of the *Snail Family Transcription Factor 1* (*SNAI1*)/*Enhancer of Zeste Homolog 2* (*EZH2)*/*STAT3* complex, which activates *STAT3* and subsequently promotes *HCAR1* transcription. Thus, exploring the regulatory mechanisms of *HCAR1* and related pathways in GC ferroptosis is of significant interest.

Based on the above background, we hypothesized that *HCAR1* regulates lipid metabolism by activating the AMP-activated protein kinase-stearoyl-CoA desaturase 1 (AMPK-SCD1) signaling pathway, which affects the ferroptosis process. Clarifying the role of *HCAR1* in GC development was the primary objective of this investigation, with particular attention paid to its effects on cell proliferation, ferroptosis control, and dysregulation of lactate-mediated metabolic pathways. Through bioinformatics analysis, we identified key prognostic genes and pathways associated with GC. Our findings demonstrate that *HCAR1* influences ferroptosis via the lactate-mediated AMPK-SCD1 axis, highlighting its value in therapeutic intervention. This research provides fresh perspectives on the metabolic regulation of ferroptosis in GC and presents new opportunities for therapeutic intervention.

## 2 Material and Methods

### 2.1 Database Acquisition

The Cancer Genome Atlas (TCGA) database (https://www.cancer.gov/tcga (accessed on 10 July 2025)) provided the genomic data for 375 STAD samples and 32 adjacent standard samples and 375 STAD samples was obtained from the Cancer Genome Atlas (TCGA) database (https://www.cancer.gov/tcga (accessed on 10 July 2025)). Gene expression profiles were retrieved from the GSE19826 dataset inside the Gene Expression Omnibus (GEO) collection (https://www.ncbi.nlm.nih.gov/gds/ (accessed on 10 July 2025)) for 12 GC tissue samples with 15 nearby standard tissue samples. Furthermore, the FerrDB database (http://www.zhounan.org/ferrdb/current/ (accessed on 10 July 2025)) yielded 395 ferroptosis-associated genes (Table S1).

#### 2.1.1 Differential Expression and Ferroptosis-Related Gene Analysis

Using the R software’s “Limma” package (version 4.1.0), differential gene expression analysis was performed on the TCGA-STAD dataset. Genes were filtered based on fold change (FC), with FC > 1.5 considered upregulated differentially expressed genes (DEGs) and FC < 0.67 considered downregulated DEGs, establishing statistical significance at *p* < 0.05. The Venn online graph tool (https://bioinformatics.psb.ugent.be/webtools/Venn/) is accustomed to analyzing the overlap between ferroptosis-related genes and between TCGA-upregulated and downregulated DEGs, and the intersection genes were filtered out for follow-up examination.

#### 2.1.2 Analysis of Functional Enrichment Analysis

The intersecting genes were subjected to enrichment analysis using the DAVID database (https://david.ncifcrf.gov/tools.jsp (accessed on 10 July 2025)), which integrates Gene Ontology (GO) and Kyoto Encyclopedia of Genes and Genomes (KEGG) pathway information. GO classification categorizes gene and protein functions into three categories: Biological Process (BP), Cell Component (CC), and Molecular Function (MF) [20].

#### 2.1.3 Analysis of the Prognostic Signature Model in GC

Using the R package “glmnet” (version 4.1.1), we applied the Least Absolute Shrinkage and Selection Operator (LASSO) Cox regression to analyze the overlapping genes obtained in this study. With ten-fold cross-validation, the ideal regularization parameter (λ) was identified, with λ.min = 0.0534 ensuring model robustness and predictive performance. The coefficients were utilized to determine the risk ratings for every patient that came from the Cox regression model with LASSO: Riskscore = (0.1041) ∗ *PHKG2* + (−0.0958) ∗ *MYB* + (0.0187) ∗ *NADPH Oxidase 4* (*NOX4*) + (−0.0019) ∗ *Solute Carrier Family 11 Member 2* (*SLC11A2*) + (−0.004) ∗ *SAT1* + (0.0069) ∗ *KRAS* + (0.0268) ∗ *PARP15* + (0.1221) ∗ *HCAR1* + (0.005) ∗ *CP* + (0.087) ∗ Aldo-*Keto Reductase Family 1 Member C2* (*AKR1C2)* + (0.0272) ∗ *LIFR*. Patients were divided into high-risk and low-risk groups according to their median risk ratings. The distribution of survival status and risk ratings among these categories was investigated. To assess the progression-free survival (PFS) probabilities across the groups, Kaplan-Meier (KM) survival curves were produced using R’s “survival” package (version 4.1.2). The risk model’s forecast precision was evaluated using ROC curves. The area under the curve (AUC) values for 1-, 3-, and 5-year survival were determined, confirming the model’s ability to forecast over different time points.

#### 2.1.4 Mutation Analysis of Prognostic Genes in GC

Mutation analysis of the 11 prognostic genes identified in our study was conducted using the Gene Set Cancer Analysis (GSCA) database, accessible at http://guolab.wchscu.cn/GSCA/#/ (accessed on 09 July 2025). This database provides comprehensive genomic data, comprising single-nucleotide variants (SNVs) as well as copy number variations (CNVs), relevant to various cancer types, including GC. First, we retrieved SNV data for the prognostic genes from the GSCA database. SNVs were analyzed to identify the frequency and types of mutations present in these genes across GC samples. Next, using the “maftools” package in R (version 4.1.0), a Spearman correlation analysis was carried out between the CNV data and the mRNA expression levels of the 11 prognostic genes. This analysis assessed the relationship between copy number alterations and gene expression, providing insights into how genomic variations might influence gene expression patterns in GC.

#### 2.1.5 Survival Analysis and Nomogram Construction for Prognostic Genes in GC Patients

Survival analysis of the overlapping prognostic genes was conducted using both univariate Cox regression (uni-Cox) and multivariate Cox regression (multi-Cox) methods. These analyses were performed using the R software, specifically utilizing the “survival” (version 4.1.2) and “forestplot” (version 4.1.2) packages for statistical computation and visualization. For the multivariate Cox regression analysis, clinical variables, along with predictive gene expression levels, were included as covariates. The “rms” tool in R (version 4.1.0) was used to create a nomogram based on the results of the multi-Cox regression. Each prognostic gene and clinical variable’s contribution to predicting the likelihood of survival for GC patients at 1, 3, and 5 years was graphically represented by the nomogram.

#### 2.1.6 Expression Analysis and Survival Evaluation of Candidate Genes in Patients with GC

The Sangerbox website (http://vip.sangerbox.com/home.html (accessed on 09 July 2025)) was utilized to examine the TCGA-STAD and GSE19826 datasets to evaluate possible gene expression levels. In order to evaluate the prognostic importance of the discovered hub genes, survival curve analysis was carried out using the GSCA database. The statistical significance of two groups is ascertained using the log-rank test. The median gene expression distribution was used to define “high expression” and “low expression” in these studies.

### 2.2 Cell Culture and Treatments

GC cell lines (AGS, HGC-27, MKN-45) and GES-1 (non-malignant gastric epithelial cell line) were provided by the Chinese Academy of Sciences’ Cell Bank in Shanghai, China. Every cell line was grown in Dulbecco’s Modified Eagle Medium (DMEM; Cat. No. 11965, Gibco, Thermo Fisher Scientific, Inc., Waltham, MA, USA), which was enhanced with 10% fetal bovine serum (FBS; cat. no. 16000044; Gibco, Thermo Fisher Scientific, Inc., Waltham, MA, USA) and 1% penicillin/streptomycin (cat. no. 15140122; Gibco, Thermo Fisher Scientific, Inc., Waltham, MA, USA). GES-1, AGS, HGC-27, and MKN-45 cells were maintained at 37°C in an atmosphere with humidity containing 5% CO_2_. The chemical reagents used in this study, including lactate, Erastin (cat. no. S7242), and Liproxstatin-1 (Lip-1, cat. no. S7699), were purchased from Selleck Chemicals (Shanghai, China). For lactate treatment, cells were incubated in DMEM with varied lactate concentrations (0, 10, 20, and 30 mM) for 24 h. Erastin and Lip-1 were diluted in Dimethyl Sulfoxide (DMSO) to 5 µM and administered to cultured cells, which were subsequently treated for 24 h. All cell lines had undergone Short Tandem Repeat (STR) identification prior to purchase and tested negative for mycoplasma contamination upon receipt.

### 2.3 Cell Transfection

Cells were cultivated at a density of 5 × 105 cells per well in 6-well plates. Incubation was continued until the cells attained 70%–80% confluence. 10 µL of Lipofectamine^TM^ 2000 (cat. no. 11668; Invitrogen, Thermo Fisher Scientific, Inc., Waltham, MA, USA) was used for transfection, following the manufacturer’s instructions. Specifically, AGS and HGC-27 cells were transfected with small interfering RNA (siRNA) targeting *HCAR1* (si-*HCAR1*) and a siRNA negative control (si-NC). In overexpression experiments, AGS and HGC-27 cells were transfected using the pcDNA3.1 expression vector (cat. no. V79020; Thermo Fisher Scientific, Inc., Waltham, MA, USA) that overexpresses the *HCAR1* gene, while NC cells were transfected using an empty vector. During transfection, a final siRNA concentration of 50 nM and a plasmid DNA concentration of 2 μg/mL were used. siRNA sequence (sense: 5^′^-UCAUCAUGGUGGUGGCAAUTT-3^′^, antisense: 5^′^-AUUGCCACCACCAUGAUGATT-3^′^) targeting HCAR1 was used. The transfection effect was verified by Quantitative Real-Time Polymerase Chain Reaction (qRT-PCR) with Western blotting (WB).

#### 2.3.1 Quantitative Real-Time Polymerase Chain Reaction (qRT-PCR)

Following the manufacturer’s instructions, total RNA was extracted from GES-1, AGS, HGC-27, and MKN-45 cells using the TRIzol reagent (cat. no. DP424; Tiangen Biotech Co., Ltd., Beijing, China). The PrimeScript RT Reagent Kit (cat. no. RR037A; Takara, Nojihigashi, Japan) was used to create complementary DNA (cDNA). Quantitative PCR was conducted with SYBR Green Master Mix (cat. no. Q121-02; Vazyme, Nanjing, China) on a StepOnePlus Real-Time PCR System (Applied Biosystems, Thermo Fisher Scientific, Inc., Waltham, MA, USA). Initial denaturation at 95°C for 5 min, 40 cycles of 95°C for 15 s, 60°C for 30 s, and 72°C for 1 min, and a final extension at 72°C for 5 min comprised the amplification technique. The internal control was GAPDH. The 2^−ΔΔCt^ technique was used to calculate relative gene expression. Table 1 contains the primer sequences.

**Table 1:** Primer sequences for qRT-PCR

Target	Direction	Sequence (5^′^-3^′^)
*HCAR1*	Forward	TTCGTATTTGGTGGCAGGCA
	Reverse	TTTCGAGGGGTCCAGGTACA
*GPX4*	Forward	ATGAGCCTCGGCCGCCTTTG
	Reverse	CCCACAAGGTAGCCAGGGGT
*SLC7A11*	Forward	CGCTGTGAAGGAAAAAGCACA
	Reverse	TGGTGGACACAACAGGCTTT
*SCD1*	Forward	AAAGCTCGGCTGCCCCTACG
	Reverse	TTGTGGTGGGCACGGTGGTC
*ACSL4*	Forward	GGCACGCGGTTCCTTTTTG
	Reverse	AGCCGACAATAAAGTACGCAA
*GAPDH*	Forward	AACACCACCATGGAGAAGGC
	Reverse	ACAGCCTTGGCAGCACCACT

#### 2.3.2 Western Blotting (WB) Assay

For total protein extraction, GES-1, AGS, HGC-27, and MKN-45 cells were treated with RIPA lysis buffer enriched with protease and phosphatase inhibitors (Beyotime, cat. no. P1046, Shanghai, China). To determine the protein content, the BCA Protein Assay Kit (cat. no. P0011; Beyotime, Shanghai, China) was utilized. Using SDS-PAGE, the protein was divided into equal portions and then applied to polyvinylidene fluoride (PVDF; cat. no. FFP39; Beyotime, Shanghai, China) membranes. To probe the membranes, primary antibodies against HCAR1 (cat. no. ab106942, 1:1000), GPX4 (cat. no. ab125066, 1:3000), SLC7A11 (cat. no. ab307601, 1:1000), SCD1(cat. no. ab19862, 1:1000), ACSL4 (cat. no. ab155282, 1:5000), AMPK (cat. no. ab222491, 1:1000), phosphorylated (p)-AMPK (cat. no. ab23875, 1:1000), SREBP1 (cat. no. ab28481, 1:5000), were used, all of which were obtained from Abcam (Cambridge, UK). The loading control for cytoplasmic proteins was GAPDH (cat. no. ab8245, 1:5000). After being incubated with HRP-conjugated secondary antibodies (Abcam, Cambridge, UK), the protein bands were detected using an ECL detection system (Beyotime, Shanghai, China) and captured on camera using ImageJ software (version 1.8.0). The expression of these proteins in cells was evaluated by WB analysis.

#### 2.3.3 Assay for Cell Counting Kit-8 (CCK-8)

The CCK-8 kit (cat. no. CK04, Dojindo Laboratories, Inc., Kumamoto Prefecture, Japan) was used to assess the viability of AGS and HGC-27 cells. Cells were seeded onto 96-well plates at a density of 5 × 10^3^ cells per well. A CCK-8 reagent was applied to each well following cell siRNA transfection or treatment with erastin (5 μM), lactate (20 mM), or Lip-1 (5 μM). After 24 h, the absorbance at 450 nm was measured using a microplate reader (Thermo Fisher Scientific, Inc., Waltham, MA, USA).

#### 2.3.4 Measurement of Reactive Oxygen Species (ROS) and Malondialdehyde (MDA)

In 96-well plates, AGS and HGC-27 cells were grown at a density of 1 × 10^4^ cells per well. Following cell apposition, the medium and unidentified cell debris were removed from the cell monolayer by twice washing it with PBS buffer (pH 7.4, 10 mM). Subsequently, lysate was added to each well, and the well plates containing the cell lysate were placed in an ultrasonic crusher for sonication to fragment the cells further. The resulting lysates were centrifuged at 10,000× *g* for 5 min to extract the supernatant. These supernatants experienced sonication and were subsequently used to assess the levels of ROS and MDA. As instructed by the manufacturer, measurements were performed using commercial assay kits. The assay kits for ROS and MDA were obtained from the Nanjing Jiancheng Bioengineering Institute (Cat. No. E004-1-1, Cat. No. A003-2-2, Nanjing, China).

#### 2.3.5 ATP Content Assay

The ATP test was performed using an ATP detection kit (cat. no. S0026; Beyotime, Shanghai, China) and following the manufacturer’s instructions. AGS and HGC-27 cells were seeded in 6-well plates at 4 × 10^5^ cells per well. After incubation, each well received 200 µL of cell lysate, which was then centrifuged at 12,000× *g* for 10 min at 4°C to remove the supernatant. The supernatant (10 µL) was added for detection after the background ATP was depleted using 100 µL of ATP detection buffer. The Infinite M200 PRO microplate reader (Tecan Group, Ltd., Zurich, Switzerland) was used to record the optical density data.

### 2.4 Statistical Analysis

Statistical analysis was conducted using R (version 4.3.3). Data are presented as mean ± SD from triplicate experiments. For comparisons among groups, one-way or two-way ANOVA was performed based on the experimental design. Post hoc analysis using Tukey’s or Bonferroni tests was conducted for multiple comparisons when ANOVA results showed significance, and differences were regarded as significant at *p* < 0.05.

## 3 Results

### 3.1 Ferroptosis-Related Gene Differential Expression and Functional Enrichment Study in GC

**Figure 1:**
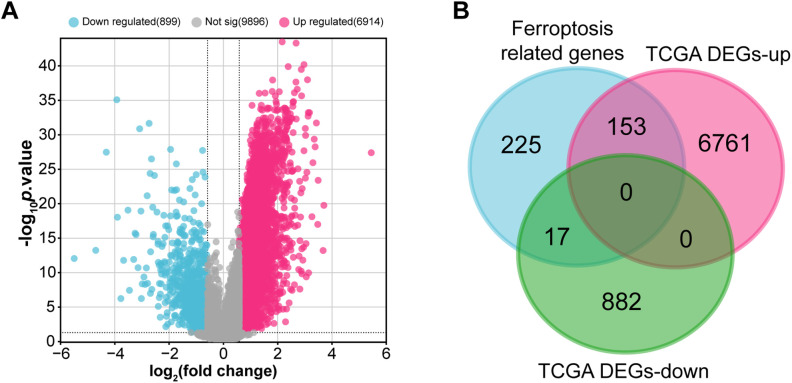

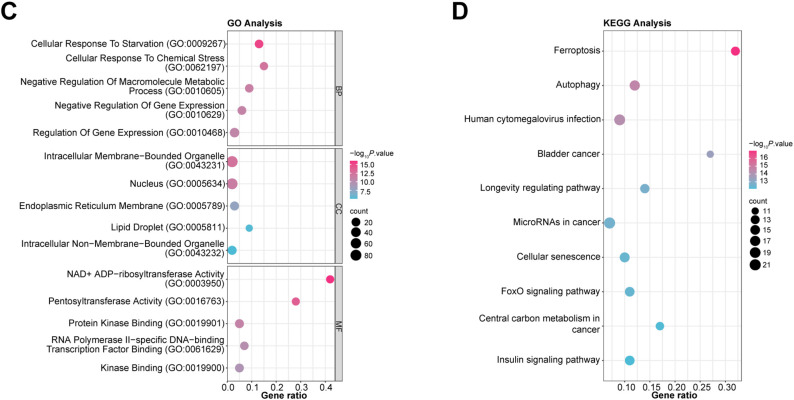
Identification and functional annotation of ferroptosis-related genes in GC. (**A**) Volcano plot of DEGs in the TCGA-STAD dataset. The *x*-axis shows the log_2_ fold change in gene expression between tumor and normal samples, while the *y*-axis represents statistical significance (−log_10_ *p*-value). Upregulated and downregulated DEGs are indicated in pink and blue, respectively. (**B**) Venn diagram illustrating the overlap among 395 ferroptosis-related genes and the DEGs. (**C**, **D**) Bubble plots summarizing GO and KEGG enrichment results for ferroptosis-associated genes. The *x*-axis indicates gene ratio; the *y*-axis shows enriched GO terms or KEGG pathways. GC, Gastric Cancer; TCGA, The Cancer Genome Atlas; STAD, Stomach Adenocarcinoma; DEGs, Differentially Expressed Genes; GO, Gene Ontology; BP, Biological Process; CC, Cell Component; MF, Molecular Function; KEGG, Kyoto Encyclopedia of Genes and Genomes

Within the TCGA-STAD cohort, 3914 upregulated and 899 downregulated DEGs were observed (Fig. 1A). Further analysis of 395 ferroptosis-related genes alongside the DEGs that are downregulated and upregulated revealed 170 intersection genes (Fig. 1B). Functional examination of the enrichment of these intersecting genes indicated significant enrichment in several BP terms, including “Cellular Response to Starvation”, “Regulation of Gene Expression”, and “Cellular Response to Chemical Stress”. Within the CC division, intersecting genes were notably enriched in terms such as “Nucleus”, “Intracellular Non-Membrane-Bounded Organelle”, and “Intracellular Membrane-Bounded Organelle”. For MF terms, significant enrichment was observed in “NAD^+^ ADP-ribosyltransferase Activity”, “Pentosyltransferase Activity”, and “Kinase Binding” (Fig. 1C). Moreover, pathway analysis using KEGG highlighted that most intersecting genes were enriched in pathways like “Ferroptosis”, “FoxO signaling pathway”, and “Insulin signaling pathway” (Fig. 1D).

### 3.2 Prognostic Significance of Potential Target Genes in GC

**Figure 2:**
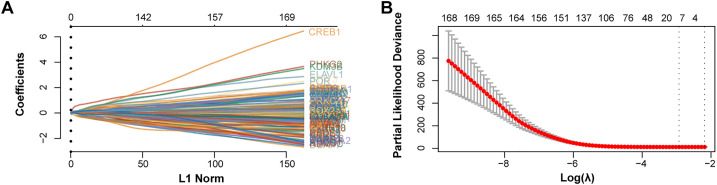

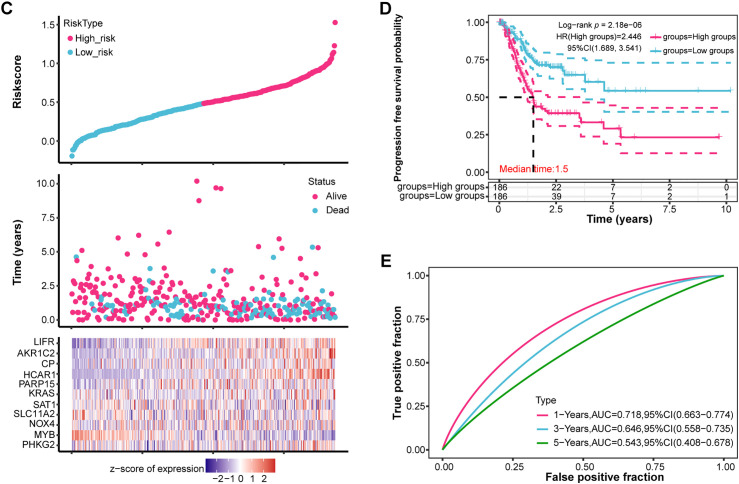
Prognostic significance of candidate genes in GC. (**A**) The coefficient spectrum was derived from LASSO regression analysis of 11 putative prognostic genes. (**B**) Tenfold cross-validated LASSO regression was utilized to optimize lambda selection. (**C**) Risk scores (**top**), patient survival outcomes (**middle**), and gene expression heatmaps (**bottom**) are visualized for the two risk groups and the four prognostic genes. (**D**) Kaplan-Meier survival curves for the four prognostic genes are shown, with time (years) on the *x*-axis and PFS probability on the *y*-axis. (**E**) Time-dependent ROC curves were generated for the risk model at 1, 3, and 5 years, and AUCs are shown using distinct colors. ROC curves of the risk model in 1 year, 3 years, and 5 years. The curves in different colors represent the AUC values in different periods. GC, Gastric Cancer; LASSO, Least Absolute Shrinkage and Selection Operator; ROC, Receiver Operating Characteristic; AUC, Area Under the Curve

Employing the LASSO Cox regression model, the study analyzed the trajectories of 170 intersecting genes as independent variables. With 10-fold cross-validation, we identified 11 potential prognostic genes [*Phosphorylase Kinase Gamma 2* (*PHKG2*), *MYB*, *NOX4*, *SLC11A2*, *SAT1*, *KRAS*, *PARP15*, *HCAR1*, *CP*, *AKR1C2*, *LIFR*] according to the optimal lambda value (lambda.min = 0.0534) as prognostic markers (Fig. 2A,B). The high-risk group had a lower survival rate and a greater death rate than the low-risk group, as seen in Fig. 2C. The high-risk group’s prognosis was further shown to be considerably worse using KM survival curves, with both groups showing a median survival time of 1.5 years (Fig. 2D). Additionally, ROC curve research revealed that the 1-year prognostic prediction accuracy was good (AUC = 0.718) (Fig. 2E). In conclusion, our study highlights the significant prognostic potential of these 11 potential prognostic genes.

### 3.3 Mutation Analysis of Potential Target Genes in GC

**Figure 3:**
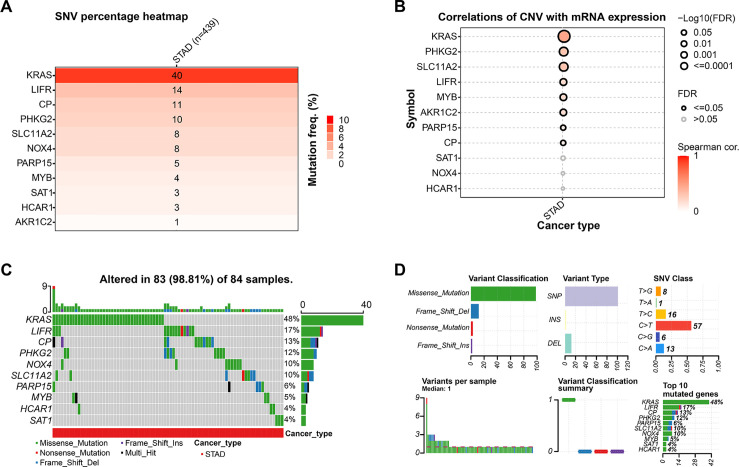
Mutation landscape of candidate genes in GC. (**A**) This heatmap shows the distribution of gene mutations in STAD, with deeper color tones representing higher mutation percentages. (**B**) Correlation analysis between CNVs and gene expression for the 11 prognostic markers in STAD, with significant correlations (*p* < 0.05) indicated. (**C**) This bar plot shows the distribution of gene mutations across 84 STAD samples. Different colors represent various types of mutations. (**D**) Variant classification summary displaying mutation types, variant types, SNV classes, and the top 10 mutated genes in the analyzed STAD samples. *x*-axis: number of mutations (**upper left**, **upper center**), Proportion of SNVs (**upper right**), Sample ID (lower left), Mutations of different functional classifications (middle and lower), Percentage of samples with mutations (**lower right**). GC, Gastric Cancer; TCGA, The Cancer Genome Atlas; STAD, Stomach Adenocarcinoma; FDR, False Discovery Rate; SNV, Single Nucleotide Variant

The mutation analysis of 11 prognostic genes in STAD, conducted through the GSCA database, revealed that *KRAS* had the most significant percentage of mutations, at 40%, followed by *LIFR* (14%) and *CP* (11%) (Fig. 3A). Using the Spearman method, CNVs were significantly positively correlated with the mRNA expression of *KRAS* along with *PHKG2* (Fig. 3B), further underscoring the relationship between genomic variation and gene expression regulation (*p* < 0.05). In 84 STAD samples, 83 (98.81%) exhibited alterations in at least one prognostic gene, with *KRAS* mutations present in 48% of the samples (Fig. 3C). Missense mutations were the predominant variant classification, and the most prevalent kind of mutation was SNP, with C > T being the most frequent SNV category. The top 10 mutated genes included *KRAS*, *LIFR*, *CP*, *PHKG2*, *NOX4*, *SLC11A2*, *PARP15*, *MYB*, *SAT1*, and *HCAR1* (Fig. 3D). Their high mutation rate in STAD may be closely associated with tumor progression and prognosis.

### 3.4 Identification of Significant Prognostic Variables in GC Using Cox Regression Models

**Figure 4:**
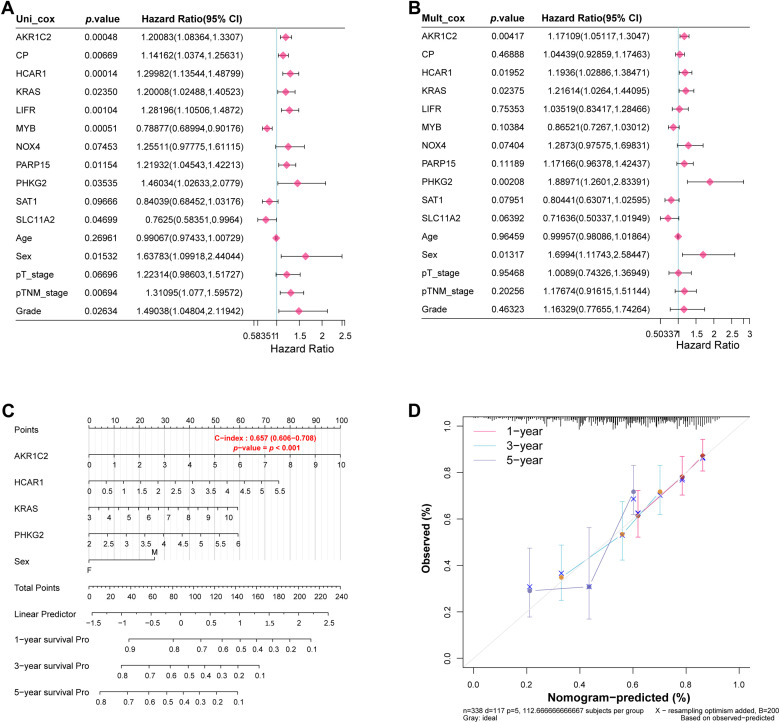
Identification of significant prognostic variables in GC. (**A**) Forest plot presenting hazard ratios (HRs) from univariate Cox regression analysis of candidate genes and clinical variables. (**B**) Forest plots show the HRs after multivariate Cox regression analysis to identify significant prognostic variables. (**C**) Based on significant prognostic factors, nomograms forecast survival rates for 1, 3, and 5 years. (**D**) Calibration curves evaluate the predictive accuracy of the nomogram for 1-year, 3-year, and 5-year survival probabilities. GC, Gastric Cancer

Through multivariate and univariate Cox regression evaluations of the 11 potential target genes and five clinical variables, we identified five statistically significant variables (*p* < 0.05): *AKR1C2*, *HCAR1*, *KRAS*, *PHKG2*, and sex (Fig. 4A,B). Nomogram analysis showed the highest prediction accuracy for 1-year survival. Calibration curves further confirmed the reliability of these predictions (Fig. 4C,D).

### 3.5 Evaluation of Gene Expression Patterns and Survival Associations in GC

**Figure 5:**
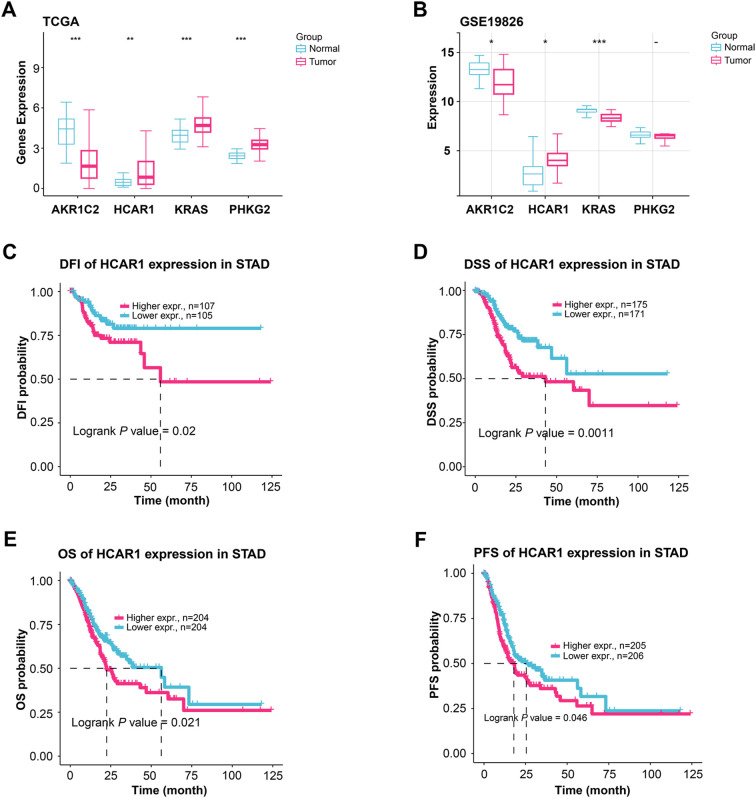
Expression patterns of key genes and their prognostic relevance in GC. (**A**, **B**) Comparing the expression levels of *AKR1C2*, *HCAR1*, *KRAS*, and *PHKG2* in tumor and normal samples from the TCGA-STAD as well as the GSE19826 dataset, comparing tumor and normal samples. (**C**–**F**) KM curves illustrate the differences in DFI (**C**), DSS (**D**), OS (**E**), and progression-free survival (PFS) (**F**) based on *HCAR1* expression levels. GC: Gastric Cancer; TCGA-STAD: The Cancer Genome Atlas-Stomach Adenocarcinoma; DFI: Disease-Free Interval; DSS: Disease-Specific Survival; OS: Overall Survival. *p < 0.05; **p < 0.01; ***p < 0.001

The study examined how four highly predictive genes in the TCGA-STAD and GSE19826 datasets (Fig. 5A,B). In the TCGA-STAD dataset, *AKR1C2* exhibited reduced expression in the tumor group; conversely, *HCAR1*, *KRAS*, and *PHKG2* showed increased expression in the tumor group. In the GSE19826 dataset, the tumor group’s expression levels of *AKR1C2* and *KRAS* declined compared to the normal group; however, *HCAR1* expression levels increased. The expression levels of *KRAS* and *PHKG2* exhibited different trends across different datasets. Conversely, elevated *HCAR1* expression correlated with unfavorable outcomes across both datasets, making *HCAR1* a more consistent and significant marker. Therefore, *HCAR1* was selected as the pivotal gene. *HCAR1* expression was associated with significantly poorer disease-free interval (DFI), disease-specific survival (DSS), overall survival (OS), and progression-free survival (PFS) than the low-expression group (Fig. 5C–F). These findings imply that a poor prognosis in GC patients may be directly associated with high *HCAR1* expression.

### 3.6 Silencing HCAR1 Inhibits GC Cell Proliferation

**Figure 6:**
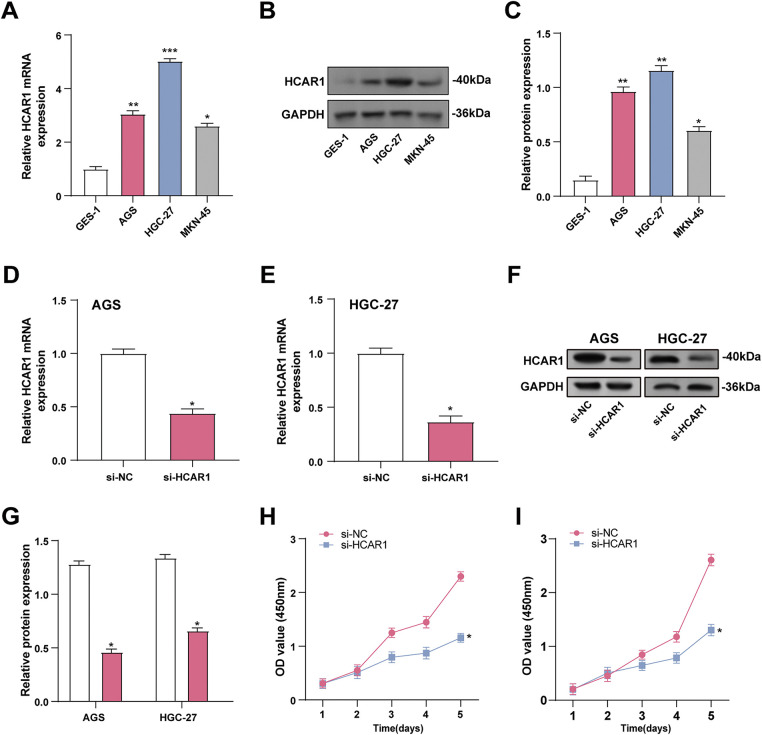
Silencing *HCAR1* inhibits GC cell proliferation. (**A**–**C**) *HCAR1* mRNA and protein levels were examined by qRT-PCR and WB in GES-1, AGS, SGC-7901, and HGC-27 cells. (**D**, **E**) The effectiveness of *HCAR1* knockdown in AGS and HGC-27 cells was tested by qRT-PCR. (**F**, **G**) WB analysis demonstrating HCAR1 protein levels in AGS and HGC-27 cells following *HCAR1* knockdown. (**H**, **I**) Quantitative analysis of CCK-8 assay results indicating cell viability following *HCAR1* knockdown. GC, Gastric Cancer; qRT-PCR, Quantitative real-time polymerase chain reaction; mRNA, messenger RNA; WB, Western blotting; CCK-8, cell counting kit-8. *p < 0.05; **p < 0.01; ***p < 0.001

*HCAR1* mRNA and protein levels were measured in GC cells and normal gastric cells (GES-1). The data indicated *HCAR1* overexpression in GC cells, with AGS and HGC-27 exhibiting the most prominent levels (Fig. 6A–C). The effectiveness of *HCAR1* knockdown was then assessed using qRT-PCR with WB techniques (Fig. 6D–G). Silencing *HCAR1* reduced AGS and HGC-27 cell viability, implying its role in promoting GC cell proliferation (Fig. 6H,I).

### 3.7 HCAR1 Upregulation Inhibits Ferroptosis in GC

**Figure 7:**
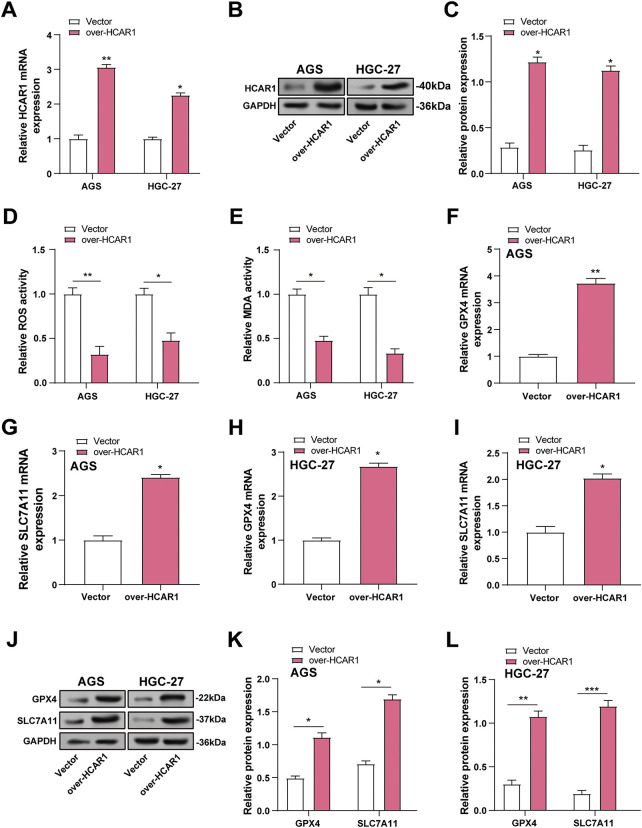
Overexpression of *HCAR1* inhibits ferroptosis in GC cells. (**A**–**C**) QRT-PCR and WB assessed expression levels of HCAR1 in AGS and HGC-27 cells overexpressing HCAR1. (**D**, **E**) In AGS and HGC-27 cells with *HCAR1* overexpression, ROS and MDA levels were quantified. (**F**–**I**) qRT-PCR was used to evaluate *GPX4* and *SLC7A11* mRNA expression in AGS and HGC-27 cells overexpressing *HCAR1*. (**J**–**L**) Upregulation of GPX4 and SLC7A11 was detected by WB in response to HCAR1 overexpression. GC, Gastric Cancer; qRT-PCR, Quantitative real-time polymerase chain reaction; mRNA, messenger RNA; WB, Western blotting; ROS, Reactive Oxygen Species; MDA, Malondialdehyde. *p < 0.05; **p < 0.01; ***p < 0.001

The efficiency of *HCAR1* overexpression in GC cells was verified by qRT-PCR and WB (Fig. 7A–C). Although ROS is essential for redox signaling, too much of it can cause oxidative stress and lipid peroxidation, which are signs of ferroptosis. MDA, a lipid peroxidation product, reflects oxidative damage. GPX4 reduces lipid peroxides, while SLC7A11 maintains cysteine for glutathione synthesis. To explore the role of *HCAR1* overexpression in regulating ferroptosis, ROS, MDA, and *GPX4*/*SLC7A11* expression were quantified in GC cells. *HCAR1* overexpression decreased ROS levels and MDA concentrations, according to assays conducted using ROS and MDA kits (Fig. 7D,E). In the results of qRT-PCR and WB analysis, *GPX4* and *SLC7A11* expressions were also upregulated after *HCAR1* overexpression compared with the control group (Fig. 7F–L). These findings imply that *HCAR1* has a function in controlling ferroptosis in GC cells.

### 3.8 Elevated Extracellular Lactate Levels Inhibit Ferroptosis in GC Cells

**Figure 8:**
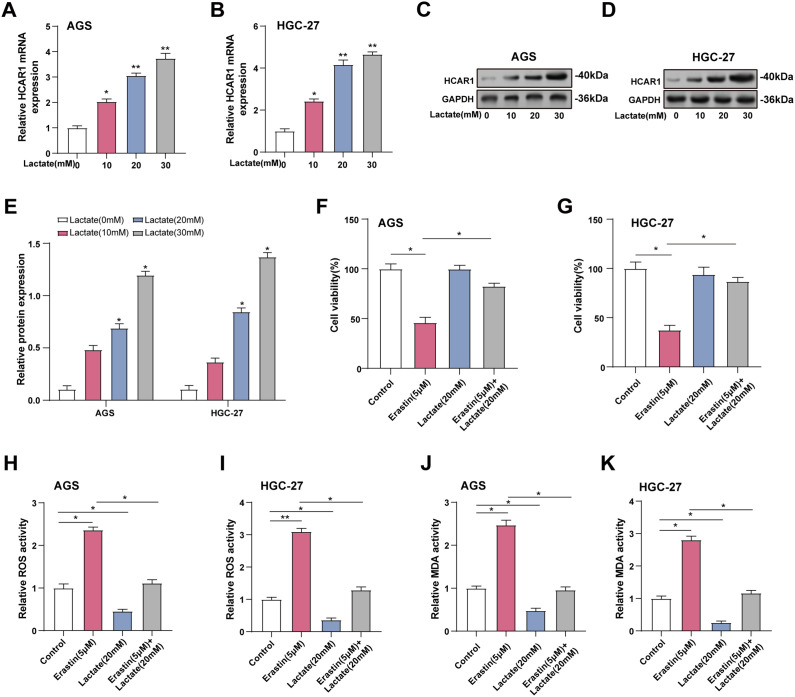

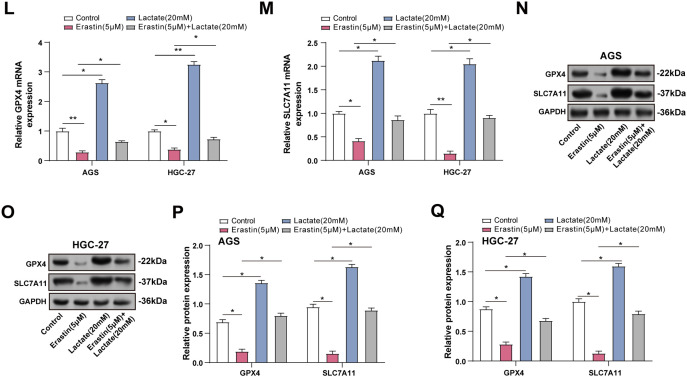
Elevated extracellular lactate inhibits ferroptosis in GC cells. (**A**, **B**) Cells treated with increasing doses of lactate (0–30 mM) were analyzed for *HCAR1* mRNA expression via qRT-PCR. (**C**–**E**) WB analysis showing *HCAR1* protein expression in AGS and HGC-27 cells treated with lactate. (**F**, **G**) CCK-8 assay was used to detect cell viability after treatment with erastin and/or lactate (**H**, **I**) Changes in ROS levels after treatment with erastin and/or lactate. (**J**, **K**) Changes in MDA levels after treatment with erastin and/or lactate. (**L**, **M**) qRT-PCR analysis of *GPX4* and *SLC7A11* mRNA expression in AGS and HGC-27 cells treated with erastin and/or lactate. (**N**–**Q**) WB analysis showing GPX4 and SLC7A11 protein expression in AGS and HGC-27 cells treated with erastin and/or lactate. GC, Gastric Cancer; qRT-PCR, Quantitative real-time polymerase chain reaction; mRNA, messenger RNA; WB, Western blotting; CCK-8, cell counting kit-8; ROS, Reactive Oxygen Species; MDA, Malondialdehyde. *p < 0.05; **p < 0.01

To mimic the increase in extracellular lactate, we assessed *HCAR1* expression in GC cells given different lactate concentrations (0, 10, 20, 30 mM) using qRT-PCR and WB. According to earlier research, lactate levels in tumor tissues are considerably higher, ranging from 10 to 40 mM, while they are modest in normal tissues, at about 1.3 mM [18,21,22]. *HCAR1* expression was elevated with increasing lactate concentration and was significantly upregulated at 20 and 30 mM (Fig. 8A–E). However, to avoid potential cytotoxicity associated with excessive lactate levels, we referenced the settings of previous studies and maintained a lactate concentration of 20 mM in subsequent experiments. We employed 5 μM Erastin, a dose adjusted in a prior investigation, to trigger iron-mediated cell death in cells to examine the impact of lactate on this process [14]. CCK-8 demonstrated that treatment with erastin reduced viability in AGS and HGC-27 cells, whereas lactate mitigated this reduction (Fig. 8F,G). In cells exposed to lactate and/or erastin, lactate was found to reduce ROS and MDA levels, while erastin therapy raised them (Fig. 8H–K). Additionally, qRT-PCR and WB were utilized to measure GPX4 as well as SLC7A11 expression in GC cells treated with lactate and/or erastin. GPX4 and SLC7A11 expression was downregulated by erastin and upregulated by lactate relative to the control group. Combined lactate and erastin treatment mitigated the erastin-induced reduction in GPX4 and SLC7A11 expression (Fig. 8L–Q). These data show that increased extracellular lactate reduces ferroptosis in GC cells by modulating antioxidant enzyme expression.

### 3.9 Inhibition of HCAR1-Promoted Lactate Transport Enhances Ferroptosis in GC Cells

**Figure 9:**
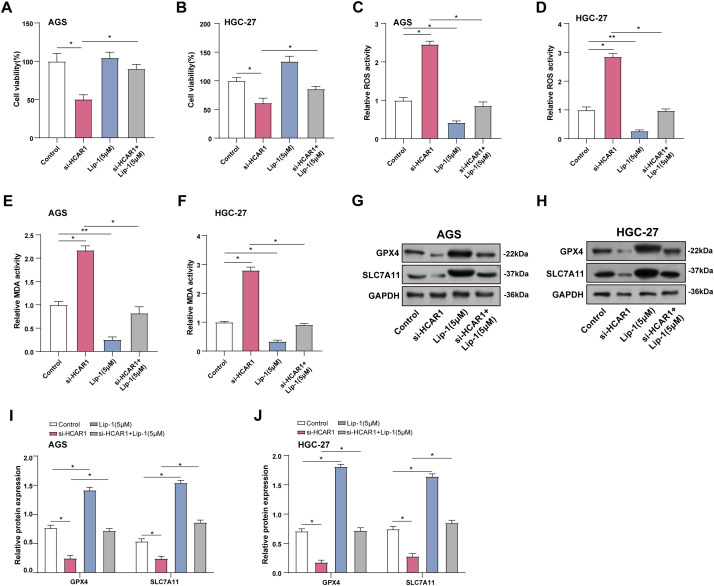
Inhibition of *HCAR1*-promoted lactate transport enhances ferroptosis in GC cells. (**A**, **B**) Viability of GC cells after *HCAR1* knockdown and Lip-1 administration was measured via CCK-8. (**C**, **D**) ROS levels were assessed in GC cells after *HCAR1* silencing and/or Lip-1 administration. (**E**, **F**) MDA concentrations were assessed in AGS and HGC-27 cells following *HCAR1* silencing and/or Lip-1 treatment. (**G**–**J**) WB analysis showing GPX4 and SLC7A11 levels in AGS and HGC-27 cells following *HCAR1* silencing and/or Lip-1 treatment. GC, Gastric Cancer; Lip-1, Liproxstatin-1; CCK-8, cell counting kit-8; ROS, Reactive Oxygen Species; MDA, Malondialdehyde; WB, Western blotting. *p < 0.05; **p < 0.01

Cell viability in GC cells was assessed using the CCK-8 assay after *HCAR1* knockdown and/or Lip-1 treatment. The outcomes demonstrated that the cell viability was severely decreased by *HCAR1* knockdown. However, the addition of Lip-1 attenuated this inhibitory effect on GC cells (Fig. 9A,B). ROS and MDA levels were measured in GC cells following *HCAR1* knockdown and/or Lip-1 treatment using specific detection kits. Compared to the control group, *HCAR1* knockdown increased ROS and MDA expression, whereas Lip-1 treatment reduced their levels. The combination of *HCAR1* knockdown and Lip-1 treatment reversed the upregulation of ROS and MDA expression after *HCAR1* knockdown (Fig. 9C–F). In addition, WB technology detected that after HCAR1 knockdown, GPX4 and SLC7A11 expression were downregulated, while Lip-1 treatment enhanced their expression. Combined treatment could reverse the effect of HCAR1 silencing (Fig. 9G–J). Lactate can also enter tumor cells via monocarboxylate transporter proteins (MCTs) [23]. To further clarify the dual role of lactate as both a signaling molecule activating HCAR1 and a metabolic substrate transported by MCTs. WB was performed to assess MCT1 expression after HCAR1 knockdown in GC cells, revealing a marked reduction in MCT1 protein levels (Fig. S1A–C). Treatment with the MCT1-specific inhibitor AZD3965 markedly reduced GC cell viability (Fig. S1D–E), and significantly elevated ROS (Fig. S1F–G) and MDA (Fig. S1H–I), whereas the ferroptosis inhibitor addition partially reversed the above changes. The above results suggest that lactate may enhance its ability to enter cells through the *HCAR1*-*MCT1* axis, thus regulating metabolism and iron-death-related processes in GC cells.

### 3.10 Lactate Induces Dysregulation of Lipid Metabolism in GC Cells

**Figure 10:**
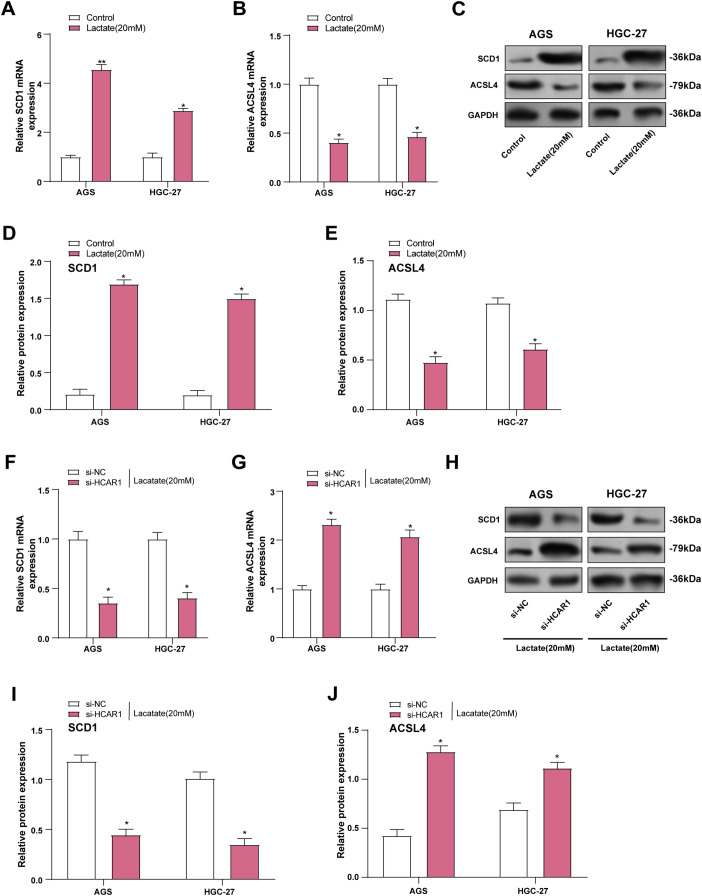
Lactate induces dysregulation of lipid metabolism in GC cells. (**A**, **B**) *SCD1* and *ACSL4* levels were assessed by qRT-PCR in lactate-treated GC cells. (**C**–**E**) WB analysis showing SCD1 and ACSL4 levels in GC cells after lactate treatment. (**F**, **G**) In GC cells, *HCAR1* knockdown-induced changes in *SCD1* and *ACSL4* mRNA were quantified using qRT-PCR. (**H**–**J**) WB analysis showing SCD1 and ACSL4 protein expression in AGS and HGC-27 cells following *HCAR1* knockdown. GC, Gastric Cancer; qRT-PCR, Quantitative real-time polymerase chain reaction; mRNA, messenger RNA; WB, Western blotting. *p < 0.05; **p < 0.01

SCD1 plays a key role in lipid metabolism by converting saturated fatty acids into monounsaturated forms [24]. Acyl-CoA synthetase long-chain family member 4 (*ACSL4*) participates in polyunsaturated fatty acid processing, which is critical for lipid signaling and the induction of ferroptosis [25]. In our study, *SCD1* expression was upregulated as well, and *ACSL4* expression was downregulated in GC cells after lactate treatment than the control group (Fig. 10A–E). Subsequently, we further examined the expression changes of both proteins after HCAR1 knockdown, and the results showed that *HCAR1* knockdown significantly downregulated SCD1 expression, while upregulating ACSL4 expression (Fig. 10F–J). To further validate the role of lactate transmembrane uptake in this process, we performed experiments with AZD3965 treatment. Western blot results showed that SCD1 expression was downregulated and ACSL4 expression was upregulated in GC cells after AZD3965 treatment (Fig. S2A–C), which was in line with the trend after HCAR1 knockdown. These results further support that lactate enters cells through the *HCAR1*-*MCT1* axis.

### 3.11 HCAR1 Regulates Ferroptosis in GC Cells via Lactate-Mediated AMPK-SCD1 Activity

**Figure 11:**
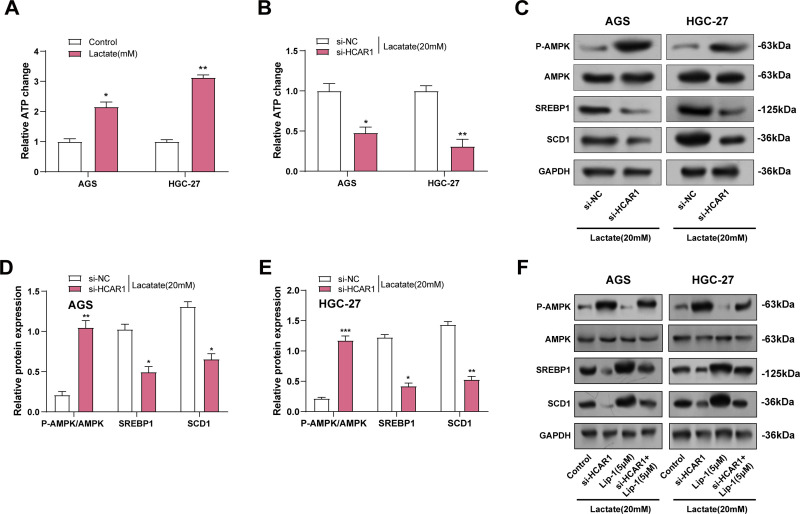

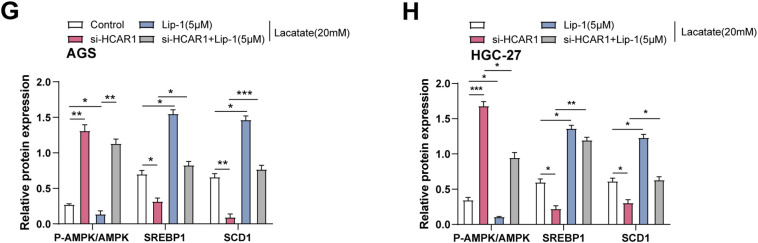
*HCAR1* regulates ferroptosis in GC cells via lactate-mediated AMPK-SCD1 activity. (**A**) Changes in ATP levels in AGS and HGC-27 cells treated with lactate were measured using ATP assay kits. (**B**) Effects of *HCAR1* knockdown on changes in ATP levels in AGS and HGC-27 cells. (**C**–**E**) WB analysis showing p-AMPK, AMPK, SREBP1, and SCD1 protein expression levels in AGS and HGC-27 cells following *HCAR1* knockdown. (**F**–**H**) Effects of *HCAR1* knockdown and Lip-1 treatment on p-AMPK, SREBP1, and SCD1 levels. GC, Gastric Cancer; ATP, Adenosine Triphosphate; WB, Western Blotting; Lip-1, Liproxstatin-1. *p < 0.05; **p < 0.01; ***p < 0.001

ATP assay kits were utilized to measure changes in ATP levels in GC cells treated with lactate. It was noted that ATP levels increased with the addition of Lactate (Fig. 11A). As depicted in Fig. 11B, ATP levels were reduced in GC cells following *HCAR1* knockdown. AMPK, a key regulator of cellular metabolism and an energy sensor, has been shown to regulate ferroptosis via altering lipid metabolism as well as oxidative stress responses [26]. To this end, we further analyzed the effects of *HCAR1* knockdown on p-AMPK, AMPK, SREBP1, and SCD1 levels by WB. HCAR1 knockdown led to a marked increase in p-AMPK expression and a reduction in SREBP1 and SCD1 protein levels (Fig. 11C–E). Furthermore, we analyzed the effects of HCAR1 knockdown and/or Lip-1 treatment, a ferroptosis inhibitor, on the above pathway proteins. The results showed that Lip-1 treatment alone decreased p-AMPK expression and up-regulated SREBP1 and SCD1 expression. In contrast, the combined treatment of *HCAR1* knockdown and Lip-1 partially reversed the changes induced by HCAR1 knockdown (Fig. 11F–H). Following treatment with the MCT1 inhibitor AZD3965 and/or Lip-1, we conducted pertinent protein expression experiments to confirm the regulatory role of lactate transport on the aforementioned pathways. WB revealed that p-AMPK expression was increased, and SREBP1 and SCD1 expression were decreased after treatment with AZD3965, and after treatment with Lip-1, p-AMPK expression decreased, and SREBP1 and SCD1 expression were upregulated after Lip-1 treatment. And after AZD3965 combined with Lip-1 treatment, p-AMPK expression was again elevated, while SREBP1 and SCD1 expression were decreased (Fig. S2D–F). These results further support the involvement of lactate in regulating the AMPK-SCD1 signaling axis and affecting the ferroptosis sensitivity of GC cells after lactate transport mediated by *HCAR1*.

## 4 Discussion

GC is still characterized by substantial morbidity and a high death rate [27]. Current research focuses on identifying novel biomarkers, elucidating their molecular mechanisms, and developing new therapeutic strategies. In our study, differential expression analysis of GC-related datasets identified 170 intersecting genes that were markedly enhanced in pathways like “Ferroptosis”, “FoxO signaling pathway”, and “Insulin signaling pathway”. Subsequent analysis identified four highly predictive genes (*AKR1C2*, *HCAR1*, *KRAS*, *PHKG2*) with prognostic potential. Liu et al. discovered that *AKR1C2* (a ferroptosis-related gene) is deregulated in GC tissues and is connected to various tumor characteristics and favorable prognosis [28]. *AKR1C2* also participates in immune-related features, underscoring its importance in GC tumorigenesis and biological function. Additionally, Yuan et al. reported that the long non-coding RNA LINC00514 is upregulated in GC, promoting cell growth and epithelial-mesenchymal transition (EMT) by inhibiting miR-204-3p and enhancing *KRAS* expression [29]. According to Geng and Wu [30], KRAS promotes GC cell growth and metabolism through ARF6, influencing the Warburg effect and oxidative stress. These effects may contribute to ferroptosis inhibition and drug resistance, highlighting KRAS as a key factor in GC development. In a study by Zhu et al. [31], *PHKG2* was found to enhance RSL3-induced ferroptosis sensitivity by regulating *ALOX5* expression in *H. pylori*-infected GC cells. Lactate acts as a paracrine and autocrine signaling molecule in the tumor microenvironment [32] through the *HCAR1* receptor. Longhitano et al. demonstrated that lactate, via its transporter MCT1 and receptor *HCAR1*, promotes the proliferation and migration of glioblastoma (GBM) cells, regulates EMT and mitochondrial function, suggesting lactate and its related pathways as potential therapeutic targets against tumor progression and recurrence [33]. Given the limited research on *HCAR1* in GC, this research aims to investigate the processes of *HCAR1* in GC.

A unique type of cell death caused by lipid peroxidation that is dependent on iron is called ferroptosis [34]. Increased intracellular free iron catalyzes the Fenton reaction, generating ROS that degrade the lipids in membranes [35]. Endale et al. highlighted the role of ROS in lipid oxidation, producing compounds such as 4-hydroxynonenal, which promote lipid peroxidation of the phospholipid bilayer and potentially trigger ferroptosis [36]. MDA is produced when polyunsaturated fatty acids (PUFAs) on cell membranes oxidize due to lipid peroxidation [37]. *GPX4* is a critical enzyme in inhibiting ferroptosis by reducing lipid peroxides to lipid alcohols, thereby protecting cell membranes from oxidative damage [38]. Research by Cui et al. revealed the essential role of *GPX4* in regulating lipid peroxidation and ferroptosis in regulatory T cells (Tregs), maintaining immune homeostasis and anti-tumor immune function [39]. Specific depletion of GPX4 in Tregs leads to elevated lipid peroxide buildup and ferroptosis, enhancing superoxide generation and IL-1β secretion upon antigen stimulation, thereby promoting TH17 cell responses. *SLC7A11*, additionally referred to as *xCT*, encodes a membrane transporter protein that regulates the balance of intracellular and extracellular cystine [40]. Lee and Roh. summarized *SLC7A11* as the light chain component of the system xc- (xCT), which transports extracellular cystine into cells to promote glutathione synthesis, maintain redox balance, and inhibit ferroptosis [41]. This study examines the effect of *HCAR1* on GC cells and ferroptosis-related markers. The findings demonstrate that destruction of *HCAR1* inhibits the proliferation of GC cells, while overexpression of *HCAR1* upregulates *GPX4* and *SLC7A11*, thereby suppressing ferroptosis. These findings underscore the interplay between ferroptosis, *HCAR1*, and GC.

Tumor cells utilize aerobic glycolysis to generate energy, resulting in the rapid accumulation of lactate [42]. This accumulation alters the tumor microenvironment, contributing to acidosis, nutrient deprivation, and immune suppression, promoting cancer cell growth, infiltration, and metastasis. Elevated lactate levels in GC patients are associated with poor prognosis, indicating that lactate detection may aid in assessing disease progression and prognosis. Wang et al. demonstrated that lactate enhances DBF4 expression by inhibiting miR-30a, playing a crucial role in tumor progression and chemotherapy sensitivity [43]. Moreover, Yang et al. found that the hypoxic tumor microenvironment enhances solid tumor resistance to ferroptosis through HIF-1α-dependent mechanisms, with HIF-1α and HIF-2α acting as major drivers of tumor resistance under hypoxic conditions [44]. Erastin primarily inhibits xCT by targeting *SLC7A11*, resulting in a decreased intracellular cystine concentration and inhibited glutathione synthesis, which sensitizes cells to oxidative stress and induces ferroptosis [45]. Li-1 prevents ferroptosis by inhibiting lipid peroxidation and reducing free oxygen radical production, thereby protecting cell membranes from lipid peroxidation damage and blocking the execution of ferroptosis [46]. This study investigated the impact of extracellular lactate levels and ferroptosis inducers on GC cells, revealing that elevated extracellular lactate levels inhibit ferroptosis in GC cells. Additionally, by examining the effects of *HCAR1* knockdown and ferroptosis inhibitors on GC cells, it was found that blocking *HCAR1*-mediated lactate influx exacerbates ferroptosis. These findings underscore the intricate relationship between lactate metabolism, ferroptosis, and *HCAR1* in GC cells, offering new insights into potential therapeutic strategies.

*SCD1* and *ACSL4* are critical regulators of lipid metabolism, playing essential roles in maintaining cellular membrane structure and function, as well as in signal transduction and energy metabolism. Wang et al. revealed that *SCD1* promotes tumor growth, migration, and resistance to ferroptosis in GC, potentially through the modulation of cancer stem-like properties and regulation of cell cycle-related proteins [47]. *ACSL4* specifically binds to polyunsaturated fatty acids (PUFAs) like arachidonic acid (AA) and docosahexaenoic acid [48,49]. Lee et al. indicated that high *ACSL4* expression sensitizes intestinal-type GC cells to ferroptosis by synthesizing AA and adrenic acid (AdA). In contrast, DNA methylation-induced inhibition confers resistance to ferroptosis in stromal-type GC cells [50]. In this study, we observed that lactate treatment increases *SCD1* expression and decreases *ACSL4* expression in GC cells. Interestingly, knocking down *HCAR1* had the opposite effects regarding the expression of *ACSL4* and *SCD1* in GC cells. Considering the role of *SCD1* as the central enzyme in monounsaturated fatty acid (MUFA) biosynthesis in humans, these findings imply that *SCD1* and similar upstream pathways may be connected to lactate-mediated resistance to ferroptosis [51].

*SREBP1* is a transcription factor primarily involved in regulating genes related to cholesterol and lipid biosynthesis [52]. Changes in the ratio of ATP to AMP activate the AMP-activated protein kinase; SREBP1 is a transcription factor primarily involved in regulating genes related to cholesterol and lipid biosynthetic pathways [53]. Changes in the ATP/AMP ratio activate the AMPK pathway within cells. AMPK activation promotes various energy-producing pathways, such as glucose uptake, glucose oxidation, and the oxidation of fatty acids, to enhance ATP synthesis, even though it inhibits energy-consuming pathways, including protein and lipid synthesis [54]. Sun et al. observed the activation of SREBP-1c in human GC tissues, promoting the expression of genes linked to fatty acid production, such as *SCD1* and *FASN* [55]. Silencing SREBP-1c restored defects in migration and invasion abilities in AGS and SGC-7901 GC cells. Zou et al. demonstrated that AMPK inhibits TGF-β1 induction by suppressing Smad3 phosphorylation and activation in GC cells [56]. Liu et al. found that in lactate-enriched hepatocellular carcinoma cells, lactate uptake via *MCT1* enhances ATP production and suppresses AMPK, thereby upregulating SREBP1 and its downstream targets, including *SCD1*, promoting MUFA production and resistance to ferroptosis [57]. Our study suggests that lactate acts as an agonist of *HCAR1* extracellularly, and activation of *HCAR1* may affect intracellular ATP production and AMPK activity. This activation may influence tumor growth and progression by regulating lactate signaling. In addition, lactate enters GC cells through the *HCAR1*-*MCT1* axis and is involved in regulating metabolism and iron-related processes. Our study demonstrates that blocking *HCAR1*-mediated lactate uptake exacerbates iron-induced cell death in GC cells, whereas *HCAR1* activation inhibits iron-induced cell death and activates antioxidant defense mechanisms. Thus, the different mechanisms of lactate action within and outside GC cells provide new insights into understanding its complex role in GC progression and therapy.

The present study provides new insights into the role of *HCAR1* in regulating iron mutation in GC cells through the lactate-HCAR1-APK-SCD1 axis. However, we should also recognize some limitations of the study. First, due to resource constraints, we were unable to perform *in vivo* validation experiments to assess the functional relevance of *HCAR1* in tumor progression and iron mutation sensitivity. Animal studies, such as xenografts or orthotopic models, are needed to confirm the therapeutic potential of targeting this axis *in vivo*. Second, although we identified *HCAR1* as a potential therapeutic target, we did not experimentally evaluate the effects of known pharmacological agonists or antagonists of *HCAR1* (e.g., 3-hydroxybutyrate or 3-chloro-5-hydroxybenzoic acid), nor did we test small-molecule inhibitors of SCD1 (e.g., A939572 or CAY10566) on the iron mutant phenotype. These remain important avenues for future translational exploration. Finally, these results were primarily obtained from AGS and HGC-27 cell lines. Therefore, further validation in additional GC subtypes or cells derived from patients is required to confirm their broader applicability.

## 5 Conclusions

This study set out to elucidate the role of *HCAR1* in GC progression, with a particular focus on its influence on ferroptosis and lipid metabolism. Our findings underscore the critical role of *HCAR1* in modulating ferroptosis in GC cells through lactate-mediated AMPK-SCD1 signaling. By identifying *HCAR1* as a significant prognostic marker, our research highlights its potential as a therapeutic target for overcoming metabolic adaptations in GC. Modulating the *HCAR1*-lactate pathway could serve as a potential approach to boost ferroptosis responsiveness and optimize therapeutic efficacy in GC. Further studies are warranted to elucidate the underlying role of *HCAR1* in lipid metabolism and its wider relevance to cancer treatment.

## Supplementary Materials



## Data Availability

The datasets used and/or analyzed in the current study are available from the corresponding authors upon reasonable request.

## Supplementary Materials

The supplementary material is available online at https://www.techscience.com/doi/10.32604/or.2025.067247/s1.
