# Supplementary material for: HCAR1 Modulates Ferroptosis in Gastric Cancer via Lactate-Mediated AMPK-SCD1 Signaling and Lipid Metabolism
Source: Oncol Res. 2025 Sep 26;33(10):3101–25. doi: 10.32604/or.2025.067247 (PMC12494118; doi:10.32604/or.2025.067247)
Supplement: Supplementary file 1 [file OncolRes-33-67247-s001.docx]

**Table S1. The ferroptosis-related genes.**

| **The ferroptosis-related genes** |
| --- |
| ABCC1 |
| ABCC5 |
| ABHD12 |
| ACADSB |
| ACO1 |
| ACSF2 |
| ACSL1 |
| ACSL3 |
| ACSL4 |
| ACVR1B |
| ADAM23 |
| AEBP2 |
| AGPAT3 |
| AGPS |
| AHCY |
| AIFM2 |
| AKR1C1 |
| AKR1C2 |
| AKR1C3 |
| AKT1S1 |
| ALOX12 |
| ALOX12B |
| ALOX15 |
| ALOX15B |
| ALOX5 |
| ALOXE3 |
| AMN |
| ANO6 |
| AR |
| ARF6 |
| ARHGEF26-AS1 |
| ARNTL |
| ASMTL-AS1 |
| ATF2 |
| ATF3 |
| ATF4 |
| ATG5 |
| ATG7 |
| ATM |
| ATP5MC3 |
| BAP1 |
| BCAT2 |
| BECN1 |
| BEX1 |
| BRD2 |
| BRD3 |
| BRD4 |
| BRD7 |
| BRDT |
| BRPF1 |
| CA9 |
| CAMKK2 |
| CARS1 |
| CAV1 |
| CBS |
| CCDC6 |
| CD44 |
| CD82 |
| CDC25A |
| CDCA3 |
| CDH1 |
| CDKN1A |
| CDKN2A |
| CDO1 |
| CGAS |
| CHAC1 |
| CHMP5 |
| CHMP6 |
| CHP1 |
| CIRBP |
| circ_0007142 |
| circ_0067934 |
| circ0097009 |
| circDTL |
| circEPSTI1 |
| circGFRA1 |
| CircIL4R |
| CircKDM4C |
| circKIF4A |
| circPSEN1 |
| CircPVT1 |
| circRHBG |
| circRHOT1 |
| circ-TTBK2 |
| CISD1 |
| CISD2 |
| CISD3 |
| CLTRN |
| COPZ1 |
| CP |
| CPEB1 |
| CREB1 |
| CREB3 |
| CREB5 |
| CS |
| CTSB |
| CYB5R1 |
| CYBB |
| CYGB |
| CYP4F8 |
| DCAF7 |
| DDR2 |
| DECR1 |
| DHODH |
| DLD |
| DNAJB6 |
| DPP4 |
| DUOX1 |
| DUOX2 |
| ECH1 |
| EGFR |
| EGLN2 |
| ELAVL1 |
| ELOVL5 |
| EMC2 |
| EPT1 |
| ETV4 |
| EZH2 |
| FABP4 |
| FADS1 |
| FADS2 |
| FAR1 |
| FBXW7 |
| FH |
| FNDC5 |
| FTH1 |
| FTL |
| FTMT |
| FURIN |
| FXN |
| FZD7 |
| G6PD |
| GALNT14 |
| GCH1 |
| GCLC |
| GDF15 |
| GJA1 |
| GLRX5 |
| GOT1 |
| GPAT4 |
| GPX4 |
| GRIA3 |
| GSK3B |
| GSTM1 |
| GSTZ1 |
| H19 |
| HCAR1 |
| HDDC3 |
| HELLS |
| HIF1A |
| HMGB1 |
| HMOX1 |
| HRAS |
| hsa_circ_0008367 |
| HSF1 |
| HSPA5 |
| HSPB1 |
| IDH1 |
| IDH2 |
| IFNA1 |
| IFNA10 |
| IFNA13 |
| IFNA14 |
| IFNA16 |
| IFNA17 |
| IFNA2 |
| IFNA21 |
| IFNA4 |
| IFNA5 |
| IFNA6 |
| IFNA7 |
| IFNA8 |
| IFNG |
| IL1B |
| IL6 |
| INTS2 |
| IREB2 |
| ISCU |
| JUN |
| KDM3B |
| KDM4A |
| KDM5A |
| KDM5C |
| KDM6B |
| KEAP1 |
| KIF20A |
| KLF2 |
| KLHDC3 |
| KRAS |
| LAMP2 |
| LCE2C |
| LCN2 |
| LIFR |
| LIG3 |
| LINC00336 |
| LINC00472 |
| LINC00618 |
| LINC01833 |
| LONP1 |
| LPCAT3 |
| LYRM1 |
| MAPK1 |
| MAPK3 |
| MAPK8 |
| MDM2 |
| MDM4 |
| MEF2C |
| MEG8 |
| METTL14 |
| MGST1 |
| MIB1 |
| MICU1 |
| MIOX |
| MIR130B |
| MIR137 |
| MIR15A |
| MIR17 |
| miR-182-5p |
| MIR18A |
| MIR190A |
| MIR214 |
| MIR27A |
| MIR302A |
| MIR324 |
| MIR375 |
| miR-378a-3p |
| MIR424 |
| MIR4443 |
| MIR5096 |
| MIR522 |
| MIR539 |
| MIR545 |
| MIR670 |
| MIR6852 |
| miR-7-5p |
| MIR761 |
| MIR9-1 |
| MIR9-2 |
| MIR9-3 |
| MLLT1 |
| MLST8 |
| MMD |
| MPC1 |
| MT1DP |
| MT1G |
| MTCH1 |
| MTDH |
| MTF1 |
| MTOR |
| MUC1 |
| MYB |
| MYCN |
| NCOA3 |
| NCOA4 |
| NDRG1 |
| NEAT1 |
| NEDD4 |
| NEDD4L |
| NF2 |
| NFE2L2 |
| NFS1 |
| NOX1 |
| NOX3 |
| NOX4 |
| NOX5 |
| NQO1 |
| NR4A1 |
| NR5A2 |
| NRAS |
| NUPR1 |
| OIP5-AS1 |
| OSBPL9 |
| OTUB1 |
| P4HB |
| PANX1 |
| PANX2 |
| PAQR3 |
| PARK7 |
| PARP1 |
| PARP10 |
| PARP11 |
| PARP12 |
| PARP14 |
| PARP15 |
| PARP16 |
| PARP2 |
| PARP3 |
| PARP4 |
| PARP6 |
| PARP8 |
| PARP9 |
| PDK4 |
| PDSS2 |
| PEBP1 |
| PEDS1 |
| PEX10 |
| PEX12 |
| PEX2 |
| PEX3 |
| PEX6 |
| PGD |
| PHF21A |
| PHKG2 |
| PIEZO1 |
| PIK3CA |
| PIR |
| PLA2G6 |
| PLIN2 |
| PML |
| POM121L12 |
| POR |
| PPARA |
| PRDX1 |
| PRDX6 |
| PRKAA1 |
| PRKAA2 |
| PRKCA |
| PROM2 |
| PTEN |
| PTPN18 |
| PTPN6 |
| PVT1 |
| QSOX1 |
| RARRES2 |
| RB1 |
| RBMS1 |
| RHEBP1 |
| RNF113A |
| RPL8 |
| RPTOR |
| RRM2 |
| SAT1 |
| SCD |
| SESN2 |
| SIAH2 |
| SIRT1 |
| SIRT3 |
| SIRT6 |
| SLC11A2 |
| SLC16A1 |
| SLC1A5 |
| SLC25A28 |
| SLC38A1 |
| SLC39A7 |
| SLC40A1 |
| SLC7A11 |
| SMAD7 |
| SMG9 |
| SMPD1 |
| SNCA |
| SOCS1 |
| SOX2 |
| SQSTM1 |
| SRC |
| SREBF1 |
| SREBF2 |
| SRSF9 |
| STAT3 |
| STING1 |
| STK11 |
| SUV39H1 |
| TAFAZZIN |
| TF |
| TFAM |
| TFAP2A |
| TFRC |
| TGFB1 |
| TGFBR1 |
| TIMM9 |
| TLR4 |
| TMBIM4 |
| TMEM161B-DT |
| TMSB4X |
| TMSB4Y |
| TNFAIP3 |
| TOR2A |
| TP53 |
| TP63 |
| TRIB2 |
| TRIM26 |
| TRIM46 |
| TSC1 |
| TTPA |
| TYRO3 |
| USP11 |
| USP35 |
| VCP |
| VDAC2 |
| WWTR1 |
| YAP1 |
| YTHDC2 |
| YY1AP1 |
| ZEB1 |
| ZFAS1 |
| ZFP36 |


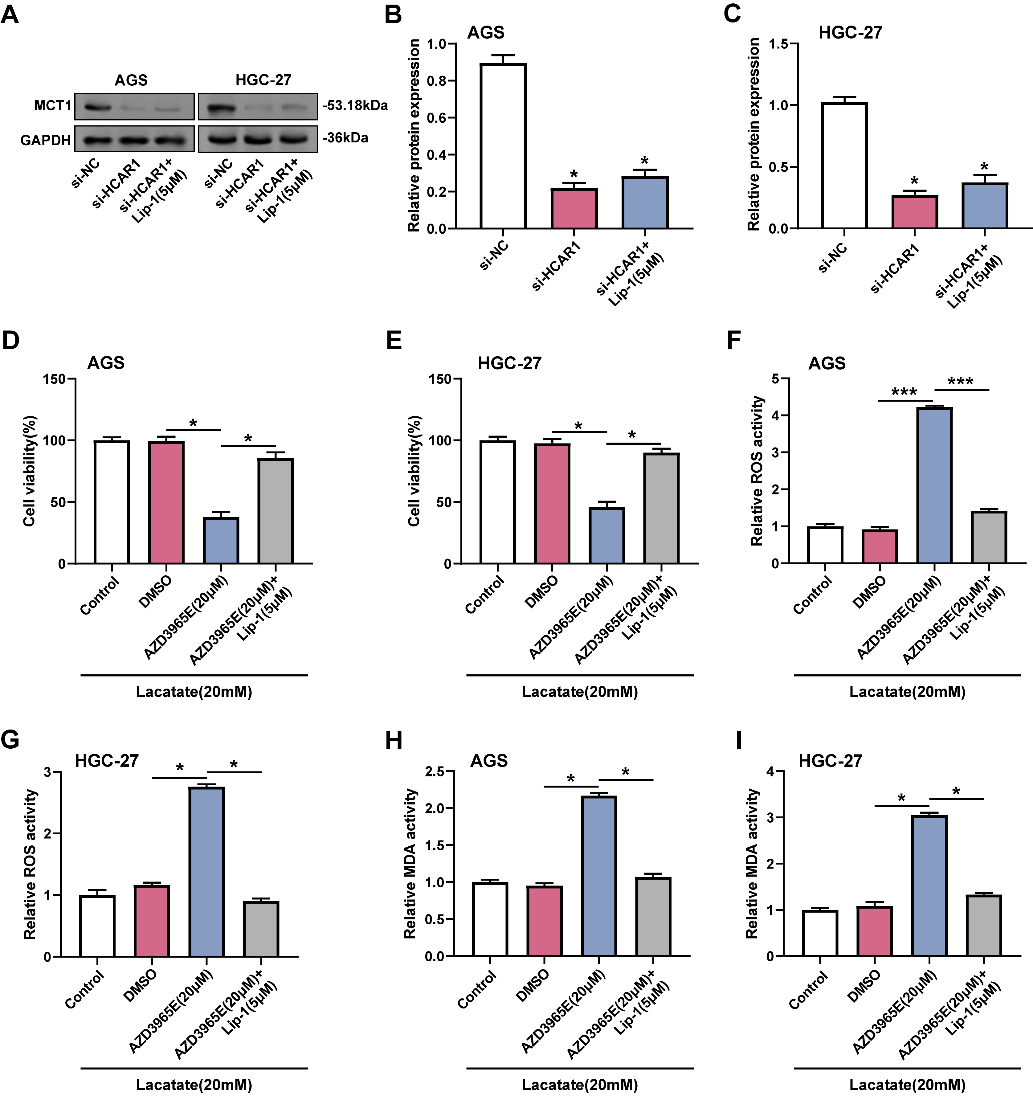


**Figure S1:** Effects of regulating HCAR1 on cellular processes in GC cells. (A) Western blot analysis of MCT1 protein expression in AGS and HGC-27 cells treated with si-NC, si-HCAR1, or si-HCAR1 plus Lip-1 (5 μM). GAPDH served as a loading control. (B and C) Quantification of MCT1 protein expression levels in AGS (B) and HGC-27 (C) cells. (D and E) Cell viability of AGS (D) and HGC-27 (E) cells treated with control, DMSO, AZD3965 (20 μM), or AZD3965 plus Lip-1 (5 μM) in the presence of 20 mM lacatate. (F and G) ROS levels in AGS (F) and HGC-27 (G) cells under the indicated treatment conditions. (H and I) MDA levels in AGS (H) and HGC-27 (I) cells under the indicated treatment conditions. GC, Gastric Cancer; Lip-1, Liproxstatin-1; CCK-8, cell counting kit-8; ROS, Reactive Oxygen Species; MDA, Malondialdehyde; WB, Western Blotting. **p* < 0.05, ****p* < 0.001.


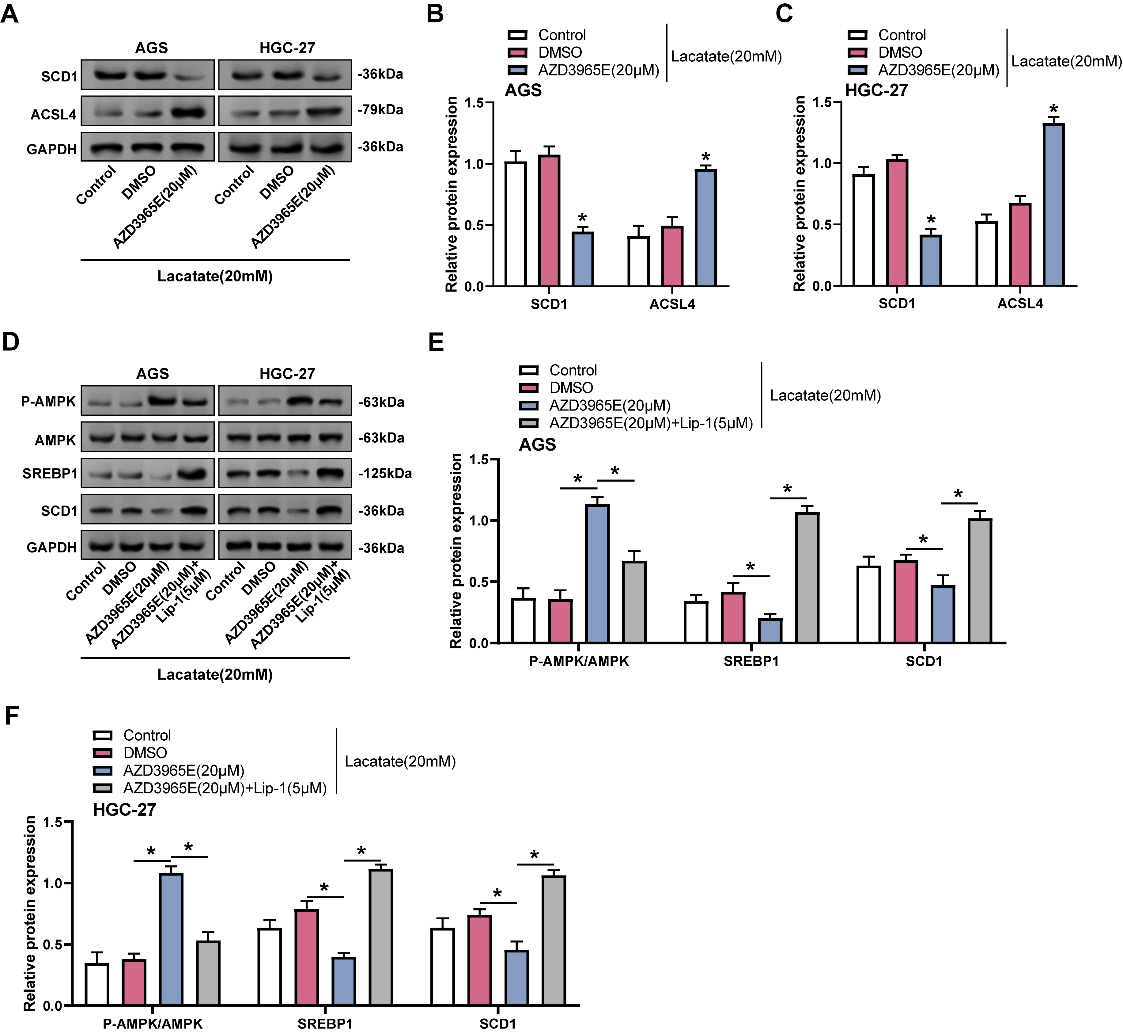


**Figure S2.** Effect of lactate on the expression of genes and proteins related to lipid metabolism in GC cells. (A) Western blot analysis of SCD1 and ACSL4 protein expression in AGS and HGC-27 cells treated with control, DMSO, AZD3965 (20 μM), or AZD3965 plus lacatate (20 mM). GAPDH served as a loading control. (B and C) Quantitative analysis of SCD1 and ACSL4 protein levels in AGS (B) and HGC-27 (C) cells. (D) Western blot analysis of phosphorylated AMPK (P-AMPK), total AMPK, SREBP1, and SCD1 protein levels in AGS andHGC-27 cells treated with AZD3965 and/or Lip-1 in the presence of 20 mM lacatate. GAPDH served as a loading control. (E and F) Quantitative analysis of P-AMPK/AMPK, SREBP1, and SCD1 protein expression in AGS (E) and HGC-27 (F) cells under the indicated treatment conditions. GC, Gastric Cancer; ATP, Adenosine Triphosphate; WB, Western Blotting; Lip-1, Liproxstatin-1. **p* < 0.05
